# Short-term effects of a new resistance exercise approach on physical function during chemotherapy after radical breast cancer surgery: a randomized controlled trial

**DOI:** 10.1186/s12905-024-02989-1

**Published:** 2024-03-05

**Authors:** Ming Huo, Xin Zhang, Jialin Fan, Hao Qi, Xuemei Chai, Minghui Qu, Yuqi Shan, Hualong Xie, Chao Wang

**Affiliations:** 1 University of Health and Rehabilitation Sciences, Qingdao City, Shandong Province China; 2 Beijing Chaoyang District Sanhuan Cancer Hospital, Beijing City, China; 3 Wangjing Hospital of China Academy of Chinese Medical Sciences, Beijing City, China

**Keywords:** Neuromuscular joint facilitation, Radical breast cancer surgery, Shoulder joint mobility disorders

## Abstract

**Background:**

Approximately 30% of post-operative breast cancer patients develop shoulder joint movement disorders affecting routine upper limb movement. This study discusses the impact of a neuromuscular joint facilitation (NJF) method on the physical function of breast cancer patients experiencing shoulder dysfunction during chemotherapy after radical surgery.

**Methods:**

This study included 162 female patients who have unilateral breast cancer in a cancer hospital in China. They developed shoulder joint mobility disorders during chemotherapy within 1–3 months postoperatively. These patients were divided into three groups: NJF, conventional rehabilitation (conventional group), and control groups. The clinical examination included the maximum passive and active range of motion (ROM) of the shoulder (flexion, extension, abduction, adduction, and external and internal rotation). Other evaluations included a pain score using a visual analog scale (VAS), grip strength, and supraspinatus muscle thickness. All tests were evaluated pre-and post-intervention.

**Results:**

The NJF group showed a significant increase in all shoulder ROM angles post-intervention. In the conventional group, all other ROM values increased significantly, except passive external rotation ROM. In the control group, all other ROM values increased significantly, except passive and active external rotation ROM. All three groups had decreased VAS scores, increased grip strength, and supraspinatus muscle thickness post-intervention during active abduction. In the control group, the supraspinatus contraction rate decreased significantly at 60° and 90° abduction post-intervention compared to that at pre-intervention.

**Conclusion:**

This study revealed that NJF during chemotherapy had positive clinical intervention effects, improving shoulder joint mobility disorders, pain, grip strength, and external rotation following radical breast cancer surgery.

**Clinical trial registration:**

Chinese Clinical Trial Registry; https://www.chictr.org.cn/ (ChiCTR2300073170), registered (03/07/2023).

## Background

Breast cancer is the most common cancer among women; it has become the second most common cancer globally and is the leading global cause of cancer-related deaths after lung cancer. According to the estimates of the International Cancer Research Center of the World Health Organization (WHO), 1.67 million new cancer cases and 525,000 deaths from breast cancer occur every year [1, 2]. The annual breast cancer economic burden in China approximates 970 million yuan [3]. The incidence of breast cancer is high among women, with a 5-year survival rate of 92%. While surgery is a radical breast cancer treatment option, it alone cannot effectively improve the prognosis. After breast cancer surgery, the most common upper limb dysfunctions include chest muscle stiffness, lymphedema, rotator cuff disease, insufficient strength, limited activity, pain, periarthritis of the shoulder, and axillary web syndrome [4]. During the 5-year follow-up after surgery, the incidence of lymphedema was 10–15%, the incidence of restricted arm/shoulder mobility was 15–30%, and the incidence of arm/shoulder pain was 30–40% [5–8].

Shoulder joint dysfunction caused by breast cancer treatment may be related to the pathological development of the affected rotator cuff [9, 10]. After breast cancer surgery, neuromuscular injury in the shoulder girdle may lead to rotator cuff disease in cancer patients [9]. The supraspinatus muscle is a vital component of the rotator cuff muscle group, playing a crucial role in stabilizing the shoulder joint. Improving the rotator cuff muscle group can enhance the motor function of the shoulder joint [11]. During the post-operative evaluation and 6-month follow-up, patients reported an increase in shoulder pain intensity and a decrease in shoulder function [12]. The trend of decreasing thickness of supraspinatus tendon on the affected side of shoulder was observed in women treated with breast cancer through ultrasound, which may lead to shoulder dysfunction of breast cancer survivors. Early detection of these possible structural changes can assist in early or preventive treatment [13].

Previous studies have demonstrated that exercise interventions for breast cancer patients can improve physical function, fatigue, depression, and quality of life. Exercise interventions include aerobic exercise, resistance training, yoga, etc. These exercises can improve muscle strength and improve quality of life [14]. Resistance training has been proved to enhance muscle function and body composition. At the same time, the risk of death of breast cancer patients undergoing resistance training has been reduced by 33% [15, 16]. Active exercise and physical therapy (stretching) benefits have previously been reported to improve joint range of motion (ROM) and post-operative pain. However, as a part of physical therapy, there are few high-quality studies on the treatment of shoulder joint dysfunction in breast cancer patients by passive movement, resistance movement, stretching, and myofascial therapy [17].

A proximal resistance neuromuscular joint facilitation (NJF) method was developed in this study. NJF is a new exercise therapy based on kinematics. This therapy combines proprioceptive neuromuscular facilitation (PNF) and joint kinematics, improving joint movement and muscle strength through passive, active, and resistance movements. NJF shoulder movement pattern is different from the traditional single axis shoulder movement training method. NJF combines the technical principle of PNF (applicable to resistance training, using muscle traction, traction and diagonal spiral motion pattern) to perform spiral diagonal motion of the upper limb. At the same time, according to the concave-convex method of joint, resistance was applied to the proximal humeral head during the movement [18]. Proximal joint resistance movement is the essential feature of this therapy. Additionally, proximal joint resistance movement can stimulate peri-capsular muscle contraction, improving limb movement function by refining joint dynamic stability during limb movement. Previous studies demonstrated that using NJF shortened electromyography reaction time, enhanced muscle strength, and improved joint flexibility and ROM [19–22]. We hypothesized that there is an improvement in the active contraction of the rotator cuff muscle by providing proximal shoulder joint resistance, thereby improving shoulder joint dysfunction after radical mastectomy.

This study aimed to explore the effect of NJF intervention on the physical function of patients with shoulder dysfunction during chemotherapy after radical cancer surgery.

## Methods

### Participants

This study was conducted at Beijing Chaoyang Sanhuan Cancer Hospital (Beijing, China) between November 2020 and March 2022. This study was approved by the Ethics Committee of the Third Ring Cancer Hospital in Chaoyang District, Beijing (registration number: ZH-2,002,013). This trial was recorded in the Chinese Clinical Trial Registry (registration date: registered (03/07/2023). registration number: (ChiCTR2300073170). All procedures were performed following the regulations and requirements of the organization. All patients or their legal guardians agreed to participate and signed an informed consent form.

This study included 162 female patients with unilateral breast cancer who developed shoulder joint mobility disorders during chemotherapy within 1–3 months postoperatively. The characteristics of the participants are presented in Table 1. These patients were randomly divided into three groups: NJF, conventional rehabilitation (conventional group), and control groups. Age, body mass index, and surgical side information were collected from all patients. Additionally, patient files were inspected to determine the surgical treatment type (modified radical mastectomy [MRM]/breast-conserving surgery [BCS] or sentinel lymph node biopsy/BCS/axillary lymph node dissection [ALND]). Body mass index (kg/m^2^) was calculated as the weight (kg) divided by height squared (meters).

**Table 1:** Participant characteristics

	NJF group (n = 51)	Conventional group (n = 50)	Control group (n = 61)	Sum total (n = 162)
Age (y)	51.3 ± 11.2	49.5 ± 10.7	50.6 ± 12.4	50.5 ± 11.5
Height (cm)	163.1 ± 4.8	161.2 ± 5.1	161.1 ± 5.3	161.7 ± 5.2
Weight (kg)	65.2 ± 8.8	62.2 ± 10.9	65.6 ± 11.0	64.4 ± 10.4

*Note* values are presented as means ± standard deviation. There were no significant differences between groups at the 0.05 level

### Sample size

The required sample number was calculated using the G*Power software. The analysis of variance (ANOVA) with repeated measures and within-between interaction methods was used. Using literature and previous case analyses, considering a 0.20 effect size and a 0.05 bilateral significance level [23, 24]. This study’s sample size was calculated using an α of 0.05 and a test efficacy (1-β) of 0.8. The minimum total sample size of 99 was calculated.

### Randomization

This study used a simple randomized method. Using the random number sequence generated by computer software, all eligible participants were randomly assigned to three groups. To eliminate bias, randomization was conducted by individuals who were not familiar with the research group. And kept in a sealed opaque envelope. The envelope can only be opened when the qualified subject agrees to enter the test, thereafter, the subjects received the corresponding intervention methods. In addition, a single blind method was used in this study. The responsible investigator will not be involved in recruitment, randomization, evaluation, or data analysis. The evaluators, and statisticians were not aware of the intervention measures for each group.

### Assessments

A physical therapist conducted the clinical examination, which included the maximum passive and active shoulder ROM during flexion, extension, abduction, adduction, and external and internal rotation. Grip strength, supraspinatus muscle thickness, and a pain score using a visual analog scale (VAS) were evaluated. Statistical analyses were performed using the mean values of the above tests pre-and post-intervention.

Based on the American Medical Association guidelines, a skilled physical therapist measured the maximum passive and active ROM during extension, abduction, adduction, and internal and external rotation using a goniometer [25].

The shoulder pain evaluation during active shoulder joint movement was scored using the VAS. A 10-centimeter line was drawn, with the left end marked as “no pain” and the right end marked as “most severe pain experienced.” Each participant marked the degree of pain pre- and post-treatment, and the distance to the leftmost end was measured.

Supraspinatus muscle thickness was measured using ultrasound (US) imaging (US; B mode, 5 MHz C5-1 transducer), a reliable method for evaluating muscle atrophy [26, 27]. A magic marker was used to indicate the 50% site above the scapular spine (linear distance from the acromial angle to the supra-scapular angle); the probe was then applied perpendicularly to the long axis of the muscle, and the longitudinal image was measured.

The US images were obtained using a probe applied with echo jelly to get clear images without compression of the superficial musculature. Supraspinatus muscle thickness was defined as the distance from the fascia at the trapezius muscle border to the scapula. Initially, patients practiced operating the US machine under the guidance of a clinical laboratory technician for approximately 1 month. The supraspinatus muscle thickness was measured at 0°, 30°, 60°, and 90° active shoulder abduction on the affected side in a sitting position. The supraspinatus muscle thickness at 0° shoulder dips was used as a reference value to calculate the supraspinatus contraction rate at 90° abduction [28, 29].

Supraspinatus contraction rate (%) = supraspinatus muscle thickness at 90° active abduction/supraspinatus muscle thickness at 0° abduction.

The upper limb grip strength of the unimpaired side was tested as a whole-body endurance indicator assessing physical and upper limb muscle strength [30]. Standard grip dynamometry was used to measure grip strength.

### Interventions

In the conventional group, all patients received four physical treatments. Physical therapy included 10 min of warming up and cooling down, including stretching and strengthening exercises. Patients performed ten replicate sets using TheraBand® for moderate-intensity shoulder flexor and abductor exercises, as well as intensive elbow flexor exercises. All exercises were conducted under the supervision of a physical therapist. Additionally, physical therapists conducted 20 min of manual treatment, including mild circular mobilization of the identified dense and hard chest wall tissue [31], as well as passive and active ROM exercises of the shoulder. Intervention frequency is once a day for four consecutive days.

In the NJF group, all patients received four physical treatments. Four NJF shoulder patterns were used: shoulder flexion-abduction-external rotation; shoulder extension-adduction-internal rotation; shoulder flexion-adduction-external rotation; and shoulder extension-abduction-internal rotation [21, 22]. Each pattern was performed three times as passive and resistance exercises. One set consisted of four NJF shoulder patterns, one set per day for four consecutive days. For patients admitted to the hospital for chemotherapy, just for a 5-day stay, the intervention was performed four consecutive times. The same physiotherapist supervised the intervention to avoid individual differences in treatment. The physical therapist who participated in the NJF technique operation in this study holds the NJF certified therapist qualification certificate organized by the Society of Physical Therapy Science and the International NJF Research Institution, and has more than five years of experience in using NJF technology.

The shoulder extension-adduction-internal rotation pattern, during NJF shoulder flexion-abduction-external rotation resistance exercises, the right hand of the physiotherapist was placed behind the right shoulder major tubercle of the patient to resist movement of the humeral head. Resistance was provided throughout the movement to the patient’s shoulder during flexion, abduction, and rotation [18]. During passive exercise, the patient was assisted with shoulder flexion-abduction-external rotation, and the left hand of the physiotherapist promoted the correct movement of the humeral head.

The shoulder extension-adduction-internal rotation pattern, during NJF shoulder extension-adduction-internal rotation resistance exercises, the left hand of the physiotherapist was placed in front of the right shoulder major tubercle of the patient to resist movement of the humeral head. Resistance was provided throughout the movement to the patient’s shoulder during extension, adduction, and internal rotation. During passive exercise, the patient was assisted with shoulder extension-adduction-internal rotation, and the left hand of the physiotherapist promoted the correct movement of the humeral head.

The shoulder flexion-adduction-external rotation pattern, during NJF shoulder flexion-adduction-external rotation resistance exercises, the left hand of the physiotherapist was placed behind the right shoulder major tubercle of the patient to resist movement of the humeral head. Resistance was provided throughout the movement to the patient’s shoulder during flexion, adduction, and external rotation. During passive exercise, the patient was assisted with shoulder flexion-adduction-external rotation, and the left hand of the physiotherapist promoted the correct movement of the humeral head.

The shoulder extension-abduction-internal rotation pattern, during NJF shoulder extension-abduction-internal rotation resistance exercises, the left hand of the physiotherapist was placed in front of the right shoulder major tubercle of the patient to resist movement of the humeral head. Resistance was provided throughout the movement to the patient’s shoulder during extension, abduction, and internal rotation. During passive exercise, the patient was assisted with shoulder extension-abduction-internal rotation, and the left hand of the physiotherapist promoted the correct movement of the humeral head.

In control groups, physical therapists did not intervene in the control group besides providing the patients with a self-training manual comprising suggestions as well as exercise methods for arms and shoulders. The manual described the correct method of active shoulder joint movement and suggested that the patient should train independently if necessary.

### Data analysis

This study used bivariate ANOVA for statistical analysis, using different group factors pre-and post-intervention. If interactions were found, univariate ANOVA and multiple comparisons (Bonferroni test) were performed using SPSS 23.0 statistical software analysis with a significance set at *p* < 0.05.

## Results

BCS and MRM were performed in 74 (45.7%) and 88 (54.3%) patients, respectively. ALND was performed in most patients (77.8%). Table 2 summarizes the patient clinical characteristics.

**Table 2:** Clinical characteristics of the study patients (n = 162)

Age, years	50.5 ± 11.5
BMI (kg/m2)	24.7 ± 3.7
Type of surgery	
BCS (+ SLNB)	28 (17.2)
BCS (+ ALND)	46 (28.4)
MRM	88 (54.3)
Type of lymph node dissection	
SLNB	36 (22.2)
ALND	126 (77.8)
Operation side	
Right	86 (53.1)
Left	76 (46.9)
Grip strength (kg)	20.3 ± 5.8
VAS	1.9 ± 1.5

Values are presented as mean ± standard deviation or n (%). BCS, breast-conserving surgery; BMI, body mass index; MRM, modified radical mastectomy; SLNB, sentinel lymph node biopsy; ALND, axillar lymph node dissection; VAS, visual analogue scale, pain severity score

Two-way ANOVA revealed interactive effects in all shoulder joint ROM angles. The NJF group demonstrated significant increases in all angles post-intervention (*p*<0.01). In the conventional group and the control group, all other ROM values increased significantly, except external rotation ROM (*p*<0.01) (Table 3).

**Table 3:** Range of motion of the shoulder joint

			NJF group (n = 51)	Conventional group (n = 50)	Control group (n = 61)	p-values for comparison between groups
Flexion	Passive	Before	138.3(29.2)	131.5(32.9)	130.9(28.5)	*P* > 0.05
		After	153.9(24.4)	152.4(23.6)	135.9(27.8)	NJF group and control group, *P* < 0.01; Conventional group and control group, *P* < 0.01.
		P-value	*P* < 0.001	*P* < 0.001	*P* < 0.01	
		95% CI	-20.29, -11.05	-25.77, 16.01	-8.12, 1.98	
	Active	Before	125.3(30.1)	124.7(26.7)	127.3(28.3)	*P* > 0.05
		After	149.0(22.6)	146.3(20.7)	132.4(28.1)	NJF group and control group, *P* < 0.01; Conventional group and control group, *P* < 0.01.
		P-value	*P* < 0.001	*P* < 0.001	*P* < 0.01	
		95% CI	-28.93, -18.63	-25.17, -17.95	-7.89, -2.25	
Extension	Passive	Before	44.1(6.4)	44.3(6.1)	43.5(7.7)	*P* > 0.05
		After	48.8(7.3)	46.9(5.3)	45.2(6.4)	NJF group and control group, *P* < 0.01.
		P-value	*P* < 0.001	*P* < 0.001	*P* < 0.01	
		95% CI	-6.88, -2.62	-3.90, -1.34	-2.85, -0.52	
	Active	Before	42.3(6.5)	43.2(7.6)	42.8(7.7)	*P* > 0.05
		After	46.4(5.2)	46.3(6.8)	44.6(7.0)	*P* > 0.05
		P-value	*P* < 0.001	*P* < 0.001	*P* < 0.01	
		95% CI	-5.66, -2.61	-4.41, -1.81	-2.85, -0.65	
Abduction	Passive	Before	136.9(32.0)	125.5(35.8)	129.2(31.0)	*P* > 0.05
		After	152.2(24.0)	149.6(29.2)	134.9(30.9)	NJF group and control group, *P* < 0.01; Conventional group and control group, *P* < 0.05.
		P-value	-20.13, -10.42	-30.24, -18.00	-8.71, -2.55	
		95% CI	*P* < 0.001	*P* < 0.001	*P* < 0.01	
	Active	Before	127.3(31.2)	118.7(33.9)	126.1(31.0)	*P* > 0.05
		After	144.1(24.2)	145.2(27.4)	132.5(30.5)	*P* > 0.05
		P-value	*P* < 0.001	*P* < 0.001	*P* < 0.001	
		95% CI	-21.43, 12.30	-32.39, 20.75	-9.61, 3.13	
Adduction	Passive	Before	39.9(6.6)	38.1(7.8)	38.3(7.0)	*P* > 0.05
		After	42.5(4.0)	42.7(3.8)	40.8(5.6)	*P* > 0.05
		P-value	*P* < 0.01	*P* < 0.001	*P* < 0.001	
		95% CI	-4.36, -0.96	-6.33, -2.95	-3.63, -1.28	
	Active	Before	38.0(8.0)	37.4(8.2)	37.7(7.4)	*P* > 0.05
		After	41.4(4.4)	42.5(3.8)	39.8(6.6)	Conventional group and control group, *P* < 0.05.
		P-value	*P* < 0.001	*P* < 0.001	*P* < 0.01	
		95% CI	-5.17, -1.67	-6.91, -3.29	-3.36, -0.91	
External rotation	Passive	Before	52.4(9.4)	53.0(9.7)	53.1(11.6)	*P* > 0.05
		After	57.1(7.4)	54.9(7.1)	53.7(7.4)	NJF group and control group, *P* < 0.05.
		P-value	*P* < 0.01	*P* > 0.05	*P* > 0.05	
		95% CI	-7.30, -2.15	-4.55, 0.59	-3.01, 1.89	
	Active	Before	50.6(10.3)	51.0(9.1)	51.4(9.6)	*P* > 0.05
		After	55.9(8.0)	54.3(7.1)	52.7(8.4)	*P* > 0.05
		P-value	P < 0.001	P < 0.01	P > 0.05	
		95% CI	-7.93, -2.64	-5.24, -1.50	-3.16, 0.43	
Internal rotation	Passive	Before	56.5(14.6)	52.9(14.4)	60.1(12.5)	Conventional group and control group, P < 0.05.
		After	61.3(9.0)	60.3(9.7)	63.4(9.2)	P > 0.05
		P-value	P < 0.001	P < 0.001	P < 0.01	
		95% CI	-7.25, -2.48	-10.05, -4.78	-5.22, -1.19	
	Active	Before	53.7(15.7)	52.2(14.3)	59.4(12.8)	Conventional group and control group, P < 0.05.
		After	60.1(9.4)	59.7(9.8)	62.5(9.5)	P > 0.05
		P-value	P < 0.001	P < 0.001	P < 0.01	
		95% CI	-9.24, -3.68	-10.13, -4.83	-5.27, -1.01	

*Note* values are presented as means (standard deviation) (unit: angle)

Before and after: p-values for comparison within groups via paired t-test

Compared with the control group: p-values for comparison between groups via Bonferroni test

NJF: neuromuscular joint facilitation; CI: confidence interval

Compared to the control group, post-intervention one-way ANOVA results revealed significantly increased passive and active flexion in the NJF and conventional groups (*p* < 0.01). Additionally, passive extension (*p* < 0.01) and external rotation (*p* < 0.05) significantly increased in the NJF group than in the control group (*p* < 0.01). Passive abduction significantly increased in the NJF (*p* < 0.01) and conventional (*p* < 0.05) groups than in the control group. Furthermore, active abduction significantly increased in the conventional group than in the control group (*p* < 0.05). However, no significant difference in active extension and external rotation was observed among the groups (Table 3).

Two-way ANOVA results demonstrated an interaction effect in the VAS (*p* < 0.01). The three groups exhibited significantly decreased VAS scores post-intervention. One-way ANOVA results revealed no significant differences between groups (Table 4).

**Table 4:** VAS and grip strength

		NJF group (n = 51)	Conventional group (n = 50)	Control group (n = 61)	p-values for comparison between groups
VAS	Before	2.18(1.44)	1.74(1.24)	1.87(1.30)	P > 0.05
	After	1.24(0.95)	1.14(0.83)	1.52(1.19)	P > 0.05
	P-value	P < 0.001	P < 0.001	P < 0.001	
	95% CI	0.663, 1.219	0.356, 0.844	0.183, 0.505	
Grip strength (kg)	Before	21.4(4.9)	19.4(4.8)	20.0(5.4)	P > 0.05
	After	23.2(4.4)	23.5(3.3)	21.0(4.7)	P > 0.05
	P-value	P < 0.001	P < 0.001	P < 0.01	
	95% CI	-2.251, -1.323	-5.039, -3.289	-1.812. -0.251	

*Note* values are presented as means (standard deviation)

NJF: neuromuscular joint facilitation; CI: confidence interval; VAS: visual analogue scale, pain severity score

p-values for comparison within groups via paired t-test

An interaction effect for grip strength was demonstrated using two-way ANOVA. All groups displayed significantly increased grip strength between pre- and post-intervention (Table 4).

Similarly, supraspinatus muscle thickness during active abduction exhibited an interaction effect using two-way ANOVA. In the NJF and conventional groups, all abduction angles were significantly increased between pre- and post-intervention. The control group exhibited significant increases in drooping position and 30° shoulder abduction between pre-and post-intervention (Table 5).

**Table 5:** Supraspinatus muscle thickness during active abduction motion of the shoulder joint

Abduction angle (°)		NJF group (n = 51)	Conventional group (n = 50)	Control group (n = 61)	p-values for comparison between groups
0°	Before	1.30(0.29)	1.29(0.26)	1.37(0.23)	P > 0.05
	After	1.42(0.24)	1.36(0.23)	1.41(0.24)	P > 0.05
	P-value	P < 0.001	P < 0.001	P < 0.01	
	95% CI	-0.156, -0.076	-0.102, -0.045	-0.059, -0.017	
30°	Before	1.47(0.29)	1.44(0.20)	1.50(0.26)	P > 0.05
	After	1.56(0.24)	1.49(0.19)	1.53(0.26)	P > 0.05
	P-value	P < 0.001	P < 0.001	P < 0.05	
	95% CI	-0.142, -0.049	-0.065, -0.036	-0.066, -0.001	
60°	Before	1.58(0.28)	1.58(0.19)	1.64(0.20)	P > 0.05
	After	1.69(0.23)	1.65(0.17)	1.66(0.22)	P > 0.05
	P-value	P < 0.001	P < 0.001	P > 0.05	
	95% CI	-0.156, -0.061	-0.098, -0.059	0.040-, 0.016	
90°	Before	1.68(0.31)	1.74(0.19)	1.74(0.24)	P > 0.05
	After	1.80(0.25)	1.81(0.18)	1.76(0.26)	P > 0.05
	P-value	P < 0.001	P < 0.001	P > 0.05	
	95% CI	-0.159, -0.078	-0.089, -0.049	-0.037, 0.009	

*Note* values are presented as means (standard deviation) (unit: cm)

NJF: neuromuscular joint facilitation; CI: confidence interval

p-values for comparison within groups via paired t-test

For the supraspinatus contraction rate, two-way ANOVA results showed an interaction effect. Supraspinatus contraction rate increased in all groups at 30° abduction post-intervention. However, the control group demonstrated a significantly decreased supraspinatus contraction rate at 60° and 90° abduction between pre- and post-intervention (Table 6).

**Table 6:** The supraspinatus muscle contraction rate during the active abduction motion of the shoulder joint

Abduction angle (°)		NJF group (n = 51)	Conventional group (n = 50)	Control group (n = 61)	p-values for comparison between groups
30°	Before	104(17)	107(11)	107(13)	P > 0.05
	After	111(13)	112(15)	109(12)	P > 0.05
	P-value	P < 0.001	P < 0.001	P < 0.05	
	95% CI	-0.110, -0.033	-0.056, -0.025	-0.044, -0.000	
60°	Before	124(19)	125(18)	122(16)	P > 0.05
	After	121(23)	124(18)	119(16)	P > 0.05
	P-value	P > 0.05	P > 0.05	P < 0.01	
	95% CI	-0.024, 0.073	-0.018, 0.043	0.007, 0.044	
90°	Before	132(23)	139(21)	130(22)	P > 0.05
	After	129(20)	136(20)	127(21)	P > 0.05
	P-value	P > 0.05	P > 0.05	P < 0.05	
	95% CI	-0.009, 0.072	-0.007, 0.070	0.006, 0.052	

*Note* values are presented as means (standard deviation) (unit: %)

NJF: neuromuscular joint facilitation; CI: confidence interval

p-values for comparison within groups via paired t-test

## Discussion

This study investigated the effect of NJF intervention on the physical function of patients with shoulder mobility disorders during chemotherapy after radical breast cancer surgery. Josenhans [32] described symptom improvement in shoulder joint ROM, pain reduction, and shoulder joint dysfunction enhancement following six physical therapy sessions. This study demonstrated that four physical therapy sessions improved shoulder joint mobility, pain score, grip strength, supraspinatus thickness, and supraspinatus distensibility in all patients. Additionally, the NJF group exhibited greater shoulder joint external rotation improvement than the conventional and control groups. This improvement greatly affected the upper limb function of patients after radical breast cancer surgery. Previous studies have shown that ROM and ROM–related strengthening exercises improved ROM during shoulder flexion, abduction, and external rotation following radical breast cancer surgery. However, shoulder abduction and external rotation showed less recovery [33]. This study demonstrated a facilitative effect on external rotation muscles because shoulder flexion-abduction-external rotation and flexion-adduction-external rotation NJF resistance movement patterns involved spiral diagonal movements and traction during resistance.

Passive movement techniques such as massage, joint mobilization, and distraction can achieve immediate results in clinical practice. However, the therapeutic effect is challenging to maintain because muscle functions, especially those supporting joint stability, such as rotator cuff muscles, do not improve post-treatment. Previous studies have shown that resistance exercise is beneficial for alleviating pain, improving lymphedema, and improving upper-limb motor function [34–36]. NJF uses proximal joint resistance for peri-articular rotator cuff muscle activation, promoting joint stability and improving intracapsular motion. Furthermore, shoulder joint NJF has previously been reported to show better improvements in shoulder mobility and pain compared to joint mobilization in patients with a frozen shoulder [37]. NJF improves upper limb motor function by relieving pain and increasing joint activity in patients with hemiplegic shoulder pain [38]. In Yoo et al.‘s 8-week water exercise study and Chae et al.‘s study, the extension and abduction angles of the intervention group increased compared to the control group, but there was no change in the external rotation angle [39]. After NJF intervention, the external rotation angle of the shoulder joint was improved. In addition, Park et al.‘s study showed that aerobic training and strength training improved the range of motion of the shoulder joint in patients with breast cancer after surgery, and the flexion angle of the shoulder joint increased by 7.66° [40, 41]. In this study, the average flexion angle of the shoulder joint increased by 15.6°. This study demonstrated the active role of NJF in shoulder function rehabilitation in breast cancer patients.

This study established the supraspinatus muscle contraction rate as a new index for shoulder dysfunction assessment. Muscle contraction rate represents muscle contraction function, elasticity, and activity, which are objective indicators for assessing muscle function. The increase in the supraspinatus contraction rate reflects increased muscle expansion and enhanced muscle contraction ability [28, 29]. Furthermore, as there may be individual differences in muscle thickness, muscle distensibility can be objectively compared between individuals eliminating the issue of individual differences. Supraspinatus contraction rate increased at 30° abduction post-intervention in all groups in this study. However, in the control group, supraspinatus contraction rate significantly decreased at 60° and 90° abduction between pre-and post-intervention, implying that supraspinatus muscle contraction capacity will reduce without active motor intervention. In the NJF group, the supraspinatus contraction rate was unchanged. This suggested that despite an improved range of motion of the shoulder joint, the function of the rotator cuff muscles would decrease without resistance proximal to the joint.

Our study has some limitations. Firstly, this study lacks an untreated control group because it is unethical for patients not to receive treatment during chemotherapy. Therefore, the natural shoulder function process in patients following radical breast cancer surgery could not be observed. Secondly, this is a short-term effect observation study; however, long-term intervention and regular follow-up are necessary. Therefore, we will conduct a long-term intervention study of NJF for more than 8 weeks in the future and follow-up the effect of the intervention to explore the long-term effect of NJF in improving shoulder dysfunction in patients with breast cancer. Data related to drugs and radiotherapy have not been collected, so it is not clear whether they may have affected the results and improved the effectiveness. Due to the limited sample size, there was no comparative analysis of different surgical types. We will discuss it in our future work. In our future work, we will include more sample sizes to further analyze the intervention effects of different surgical types. In addition, we will collect data related to drugs and radiotherapy, understand the impact of surgical types, drugs, and radiotherapy on the intervention effects, and raise the rigor of the research.

## Conclusions

In conclusion, this study suggests that NJF treatment is superior to traditional rehabilitation treatment. NJF has positive clinical intervention effects in improving shoulder joint mobility disorders, pain, grip strength, and external rotation movement of the shoulder joint during chemotherapy after radical breast cancer surgery. NJF treatment demonstrates rapid and effective alleviation of shoulder joint dysfunction in breast cancer patients, thereby enhancing upper limb motor ability. Moreover, the standardized operating pattern of NJF facilitates therapists’ proficiency and promotes its clinical applicability.

## Data Availability

The data of this research result can be obtained from the corresponding author according to reasonable requirements.

## Abbreviations

NJF: Neuromuscular joint facilitation
ROM: Range of motion
VAS: Visual analog scale
WHO: World Health Organization

## References

[1] Linghu R, Si W, Li Y (2015). Epidemiological and clinicopathological analysis of 3846 cases of breast cancer. J PLA Med Coll.

[2] Najafi S, Sadeghi M, Abasvandi F, Shajari MR, Mohebi K, Ghandchi H (2019). Prognostic factors influencing prognosis in early breast cancer patients. Prz Menopauzalny.

[3] Wang L, Yue X, Shi J (2017). Research on the economic burden of breast cancer in China in the past 20 years. Prev Control Chronic Dis China.

[4] Hidding JT, Beurskens CH, van der Wees PJ, van Laarhoven HW, Nijhuis-van der Sanden MW (2014). Treatment related impairments in arm and shoulder in patients with breast cancer: a systematic review. PLoS ONE.

[5] Norman SA, Russel Locario A, Potashnik SL, Simoes Torpey HA, Kallan MJ, Weber AL (2009). Lymphedema in breast cancer survivors: incidence, degree, time course, treatment, and symptoms. J Clin Oncol.

[6] Nesvold IL, Dahl AA, Løkkevik E, Mengshoel AM, Fosså SD (2008). Arm and shoulder morbidity in breast cancer patients after breast-conserving therapy versus mastectomy. Acta Oncol.

[7] Peuckmann V, Ekholm O, Rasmussen NK, Groenvold M, Christiansen P, Møller S (2009). Chronic pain and other sequelae in long-term breast cancer survivors: nationwide survey in Denmark. Eur J Pain.

[8] Nesvold IL, Reinertsen KV, Fosså SD, Dahl AA (2011). The relation between arm/shoulder problems and quality of life in breast cancer survivors: a cross-sectional and longitudinal study. J Cancer Surviv.

[9] Ebaugh D, Spinelli B, Schmitz KH (2011). Shoulder impairments and their association with symptomatic rotator cuff disease in breast cancer survivors. Med Hypotheses.

[10] Mafu TS, September AV, Shamley D (2018). The potential role of angiogenesis in the development of shoulder pain, shoulder dysfunction, and lymphedema after breast cancer treatment. Cancer Manag Res.

[11] Assila N, Duprey S, Begon M (2021). Glenohumeral joint and muscles functions during a lifting task. J Biomech.

[12] Torres Lacomba M, del Mayoral O, Coperias Zazo JL, Gerwin RD, Goñí AZ (2010). Incidence of myofascial pain syndrome in breast cancer surgery: a prospective study. Clin J Pain.

[13] Gala-Alarcón P, Prieto-Gómez V, Bailón-Cerezo J, Yuste-Sánchez MJ, Arranz-Martín B, Torres-Lacomba M (2021). Changes in shoulder outcomes using ultrasonographic assessment of breast cancer survivors: a prospective longitudinal study with 6-month follow-up. Sci Rep.

[14] Wang TC, Chen PL, Liao WC, Tsai IC (2023). Differential Impact of exercises on Quality-of-life improvement in breast Cancer survivors: A Network Meta-Analysis of Randomized controlled trials. Cancers (Basel).

[15] Strasser B, Steindorf K, Wiskemann J, Ulrich CM (2013). Impact of resistance training in cancer survivors: a meta-analysis. Med Sci Sport Exerc.

[16] Hardee JP, Porter RR, Sui X, Archer E, Lee I-M, Lavie CJ et al. The effect of resistance exercise on all-cause mortality in cancer survivors. Mayo Clin Proc. 2014;89:1108–1115. 10.1016/j.mayocp.2014.03.018 PMC4126241 24958698

[17] De Groef A, Van Kampen M, Dieltjens E, Christiaens MR, Neven P, Geraerts I (2015). Effectiveness of postoperative physical therapy for upper-limb impairments after breast cancer treatment: a systematic review. Arch Phys Med Rehabil.

[18] Ipec Press. Huo M [internet]. Neuromuscular joint facilitation press release. Ipec Press website. Available from: https://ipecpress.com/neuromuscular-joint-facilitation-press-release/. (accessed May 2 2023).

[19] Huo M, Ge M, Li D, Huang Q, Maruyama H (2012). Effects of neuromuscular joint facilitation on electromechanical reaction time of rectus femoris. J Phys Ther Sci.

[20] Huo M, Wang HD, Ge M, Huang QC, Li DS, Maruyama H (2013). The immediate effect of neuromuscular joint facilitation (NJF) treatment on electromechanical reaction times of hip flexion. J Phys Ther Sci.

[21] Huo M, Li D, Ge M, Huang Q, Maruyama H (2012). Effects of Neuromuscular Joint Facilitation on Electromechanical Reaction Times of the Teres Major. J Phys Ther Sci.

[22] Wu P, Huo M, Maruyama H (2013). Effects of neuromuscular joint facilitation on baseball pitching velocity and electromechanical reaction times of the teres major of young amateur baseball players. J Phys Ther Sci.

[23] Kang H (2021). Sample size determination and power analysis using the G*Power software. J Educ Eval Health Prof.

[24] Faul F, Erdfelder E, Lang AG, Buchner A (2007). G*Power 3: a flexible statistical power analysis program for the social, behavioral, and biomedical sciences. Behav Res Methods.

[25] AMA guides (1993). To the evaluation of permanent impairment.

[26] Kretić D, Turk T, Rotim T, Šarić G (2018). Reliability of ultrasound measurement of muscle thickness in patients with supraspinatus tendon pathology. Acta Clin Croat.

[27] Xie H, Lu K, Lyu G, Kang G, Huang Q, Liu S (2020). Reliability of ultrasonographic measurement of the supraspinatus thickness at different angles of shoulder abduction in patients with stroke. J Phys Ther Sci.

[28] Fukunaga T, Miyatani M, Tachi M, Kouzaki M, Kawakami Y, Kanehisa H (2001). Muscle volume is a major determinant of joint torque in humans. Acta Physiol Scand.

[29] Abe T, Loenneke JP, Thiebaud RS (2015). Morphological and functional relationships with ultrasound measured muscle thickness of the lower extremity: a brief review. Ultrasound.

[30] España-Romero V, Ortega FB, Vicente-Rodríguez G, Artero EG, Rey JP, Ruiz JR (2010). Elbow position affects handgrip strength in adolescents: validity and reliability of Jamar, Dynex, and TKK dynamometers. J Strength Cond Res.

[31] Harris R, Piller N (2003). Three case studies indicating the effectiveness of manual lymph drainage on patients with primary and secondary lymphedema using objective measuring tools. J Bodyw Mov Ther.

[32] Josenhans E (2007). Physiotherapeutic treatment for axillary cord formation following breast cancer surgery. Pt_Zeitschrift für Physiotherapeuten.

[33] Ribeiro IL, Moreira RFC, Ferrari AV, Alburquerque-Sendín F, Camargo PR, Salvini TF (2019). Effectiveness of early rehabilitation on range of motion, muscle strength and arm function after breast cancer surgery: a systematic review of randomized controlled trials. Clin Rehabil.

[34] Park JH (2017). The effects of complex exercise on shoulder range of motion and pain for women with breast cancer-related lymphedema: a single-blind, randomized controlled trial. Breast Cancer.

[35] Zengin Alpozgen A, Razak Ozdincler A, Karanlik H, Yaman Agaoglu F, Narin AN. Effectiveness of Pilates-based exercises on upper extremity disorders related with breast cancer treatment. Eur J Cancer Care (Engl). 2017;26. 10.1111/ecc.12532. 10.1111/ecc.12532 27339709

[36] Lin Y, Chen Y, Liu R, Cao B (2023). Effect of exercise on rehabilitation of breast cancer surgery patients: a systematic review and meta-analysis of randomized controlled trials. Nurs Open.

[37] Yin LQ, Yin K, Hao XB, Fan XL (2010). 30 cases of periarthritis treated by neuromuscular joint facilitation. Chin J Gerontol.

[38] Wei YH, Du DC, Jiang K (2019). Therapeutic efficacy of acupuncture combined with neuromuscular joint facilitation in treatment of hemiplegic shoulder pain. World J Clin Cases.

[39] Yoo YS (1996). Effects of aquatic exercise program on the shoulder joint function, immune response and emotional state in postmastectomy patients. J Cath Med Coll.

[40] Chae YR, Choe MA (2001). Effects of exercise on cardiopulmonary functioning in breast cancer patients undergoing radiation therapy after breast surgery. J Korean Acad Nurs.

[41] Park JH (2017). The effects of complex exercise on shoulder range of motion and pain for women with breast cancer-related lymphedema: a single-blind, randomized controlled trial. Breast Cancer.

